# Supplementary material for: Targeting the IRE1α/XBP1s pathway suppresses CARM1-expressing ovarian cancer
Source: Nat Commun. 2021 Sep 7;12:5321. doi: 10.1038/s41467-021-25684-3 (PMC8423755; doi:10.1038/s41467-021-25684-3)
Supplement: Supplementary file 1 — Supplementary Information [file 41467_2021_25684_MOESM1_ESM.pdf]

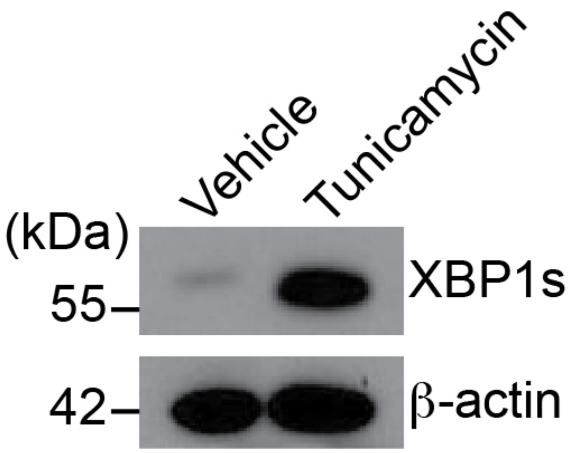

Supplementary Figure 1. CARM1 regulates XBP1s target gene expression
Expression of XBP1s and a loading control β-actin in A1847 cells treated with vehicle control or tunicamycin (5 μg/ml, 8h). Immunoblots are representative of three biologically independent experiments with similar results.

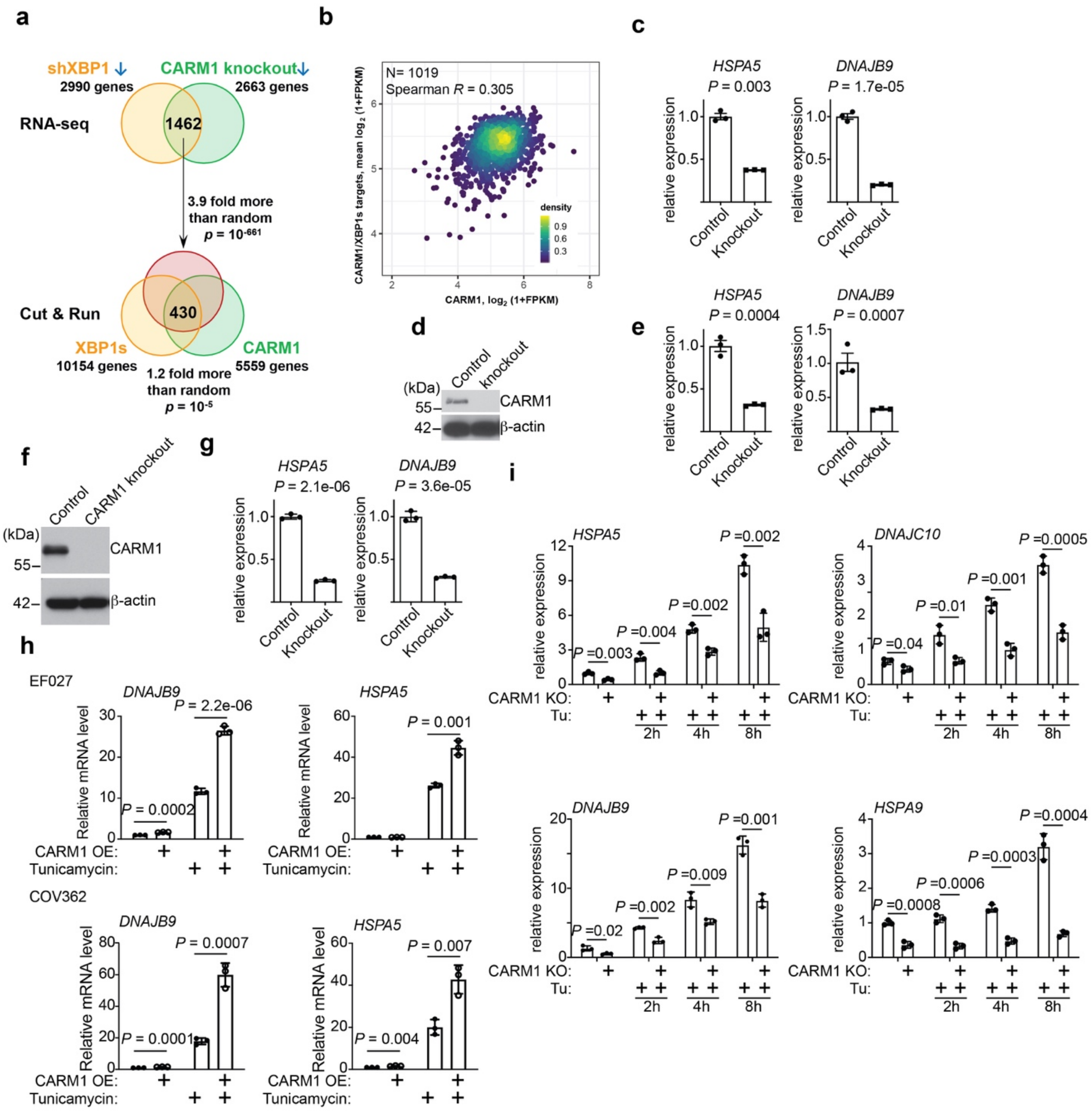

Supplementary Figure 2. CARM1 promotes XBP1s target gene expression

a, Schematic of the strategy used to identify CARM1 and XBP1s target genes under normal condition. **b,** Correlation between the expression of CARM1 and CARM1/XBP1s direct target genes in the Broad Institute Cancer Cell Line Encyclopedia database. *P* values were calculated by two-tailed Spearman R analysis. **c**, RT-qPCR analysis of XBP1s target genes *HSPA5* and *DNAJB9* in control and CARM1 knockout A1847 cells. Data represent mean ± SEM, n = 3 biologically independent experiments. *P* values were calculated using a two-tailed t test. **d**, Expression of CARM1 and a loading control β-actin in control or CARM1 knockout PEO4 cells determined by immunoblot. Immunoblots are representative of two biologically independent experiments with similar results. **e**, RT-qPCR analysis of XBP1s target genes *HSPA5* and *DNAJB9* in control and CARM1 knockout PEO4 cells. Data represent mean ± SEM, n = 3 biologically independent experiments. *P* values were calculated using a two-tailed t test. **f-g**,

Same as d-e but for OVSAHO cells. Data represent mean ± SEM, n = 3 biologically independent experiments. *P* values were calculated using a two-tailed t test. **h**, RT-qPCR analysis of XBP1s target genes *HSPA5* and *DNAJB9* in the indicated control and CRISPR-mediated CARM1 overexpressing EF027 and COV362 cells. Data represent mean ± SEM, n = 3 biologically independent experiments. *P* values were calculated using a two-tailed t test. **i**, RT-qPCR results showing the expression of the indicated CARM1/XBP1s target genes in control and CARM1 knockout PEO4 cells treated with vehicle control or tunicamycin (5μg/ml, 8h). Data represent mean ± SEM, n = 3 biologically independent experiments. *P* values were calculated using a two-tailed t test.

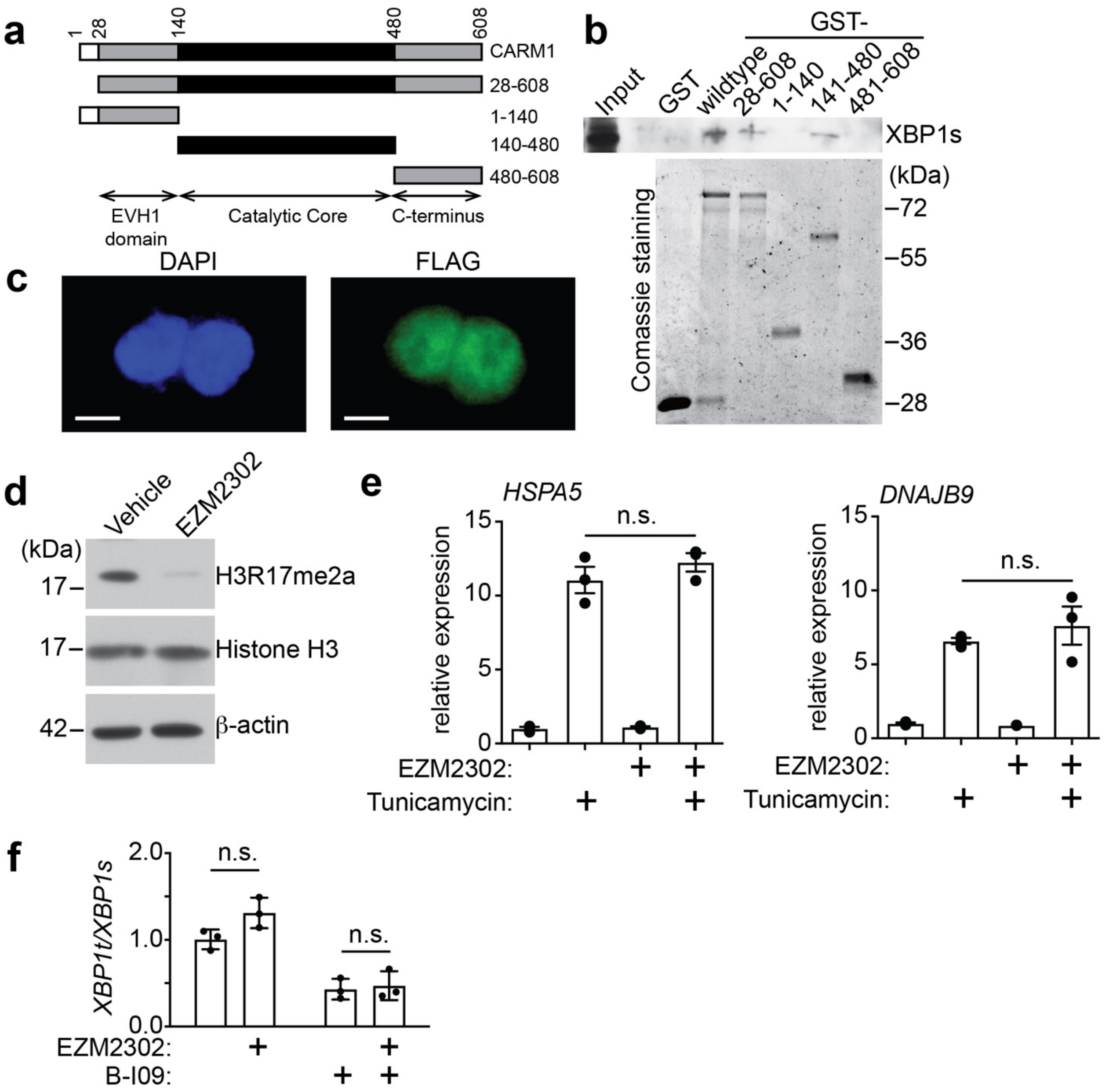

Supplementary Figure 3. XBP1s interacts with catalytic domain of CARM1
a, Diagram of truncation mutants of CARM1. **b**, GST-pull down assay using GST-tagged CARM1 truncation mutants to map the XBP1s interaction domain in CARM1. Please note that in addition to GST, GST-1-140 and GST481-608 were used as additional controls. Immunoblots are representative of three biologically independent experiments with similar results. **c**, FLAG-tagged CARM1 141-480 localized to nuclei determined by immunofluorescence staining. DAPI counter staining was used to visualize cell nuclei. Immunoblots are representative of two biologically independent experiments with similar results. Scale bar = 10 μm. **d**, Expression of H3R17me2a, an enzymatic product of CAMR1 methyltransferase activity, and loading controls histone H3 and β-actin in A1847 cells treated with vehicle control or CARM1 enzymatic inhibitor EZM2302 (20 μg/ml, 48 h) determined by immunoblot. Immunoblots are representative of two biologically independent experiments with similar results. **e**, RT-qPCR analysis of the

expression of *HSPA5* and *DNAJB9* in A1847 cells treated with vehicle control or CARM1 inhibitor EZM2302. Data represent mean ± SEM, n = 3 biologically independent experiments. *P* values were calculated using a two-tailed t test. **f**, Ratio of total and spliced XBP1 (XBP1t/XBP1s) mRNA determined by RT-qPCR analysis in the indicated A1847 cells treated with vehicle control or CARM1 inhibitor EZM2302 (20 μg/ml) with or without B-I09 treatment (5 μg/ml) for 48 h. Data represent mean ± SEM, n = 3 biologically independent experiments. *P* values were calculated using a two-tailed *t* test. n.s.: not significant.

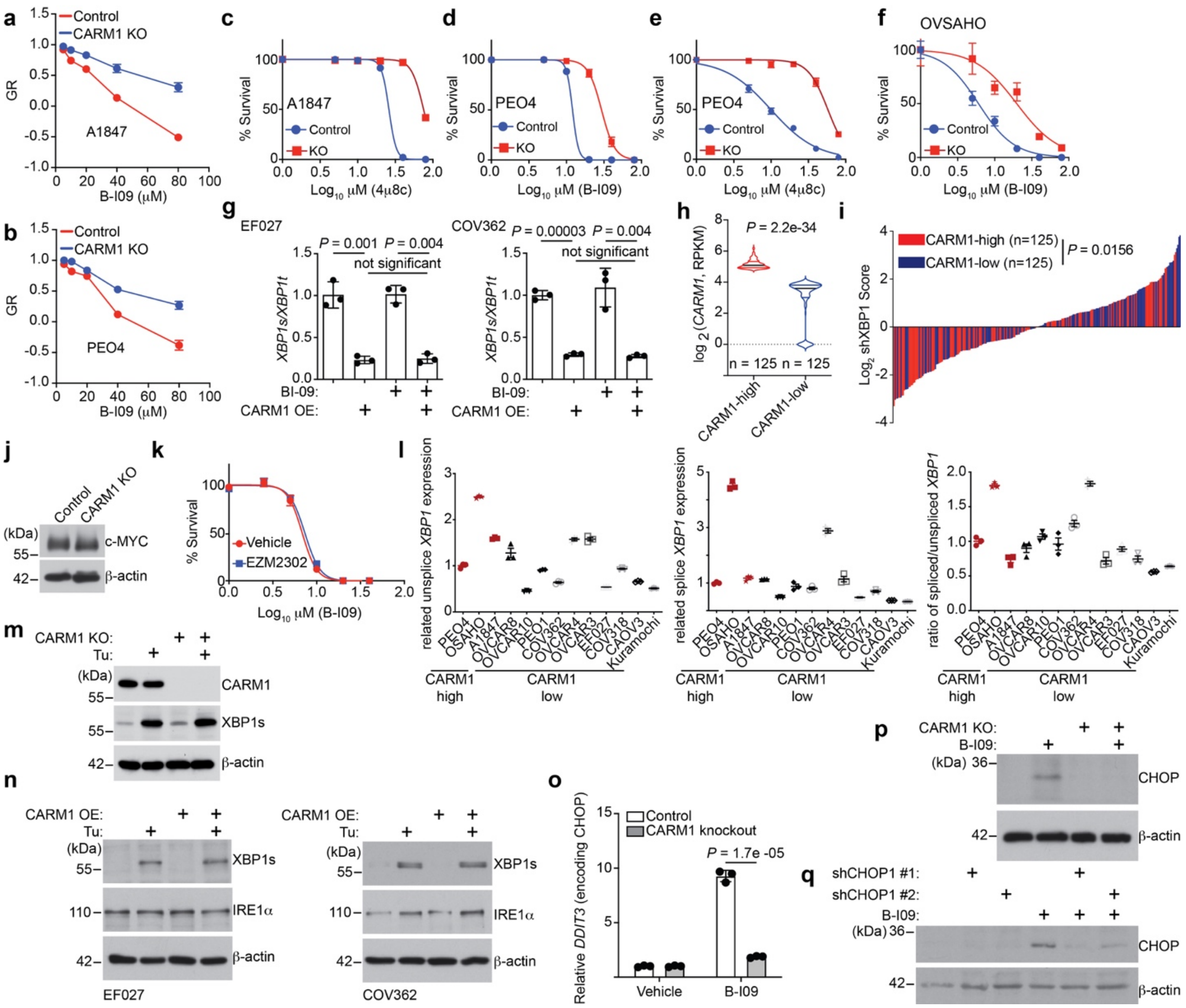

Supplementary Figure 4. CARM1 expression correlates with response to IRE1α inhibitor B-I09

a-b, Growth rate (GR) inhibition curves for the indicated control and CARM1 knockout A1847 (a) or PEO4 (b) cells treated with the indicated concentrations of B-I09 for 4 days. **c,** 4μ8c dose responsive curves for control and CARM1 knockout A1847 cells determined by colony formation assay. Data represent mean ± SEM, n = 4 biologically independent experiments. *P* values were calculated using a two-tailed *t* test. **d-e**, B-I09 (**d**) and 4μ8c (**e**) dose responsive curves for control and CARM1 knockout PEO4 cells determined by colony formation assay. **f,** B-I09 dose responsive curves for control and CARM1 knockout OVSAHO cells determined by colony formation assay. Data represent mean ± SEM, n = 4 biologically independent experiments. *P* values were calculated using a two-tailed *t* test. **g**, The ratio of spliced and unspliced *XBP1* was calculated in the indicated cells. n = 3 biologically independent experiments. *P* values were calculated using a two-tailed *t* test. **h-i**, Violin plots for expression of *CARM1* in the top quartile (CARM1-high) and low quartile (CARM1-low) cell lines in the Project Achilles database (**h**). Violin area covers whole data range, the center (black line) is represented by the median, the upper and lower lines represent quartiles (25th percentile and 75th percentile). *P* values were calculated using a two-tailed *t* test. shRNA scores in CARM1-high and CARM1-low cell lines in

the database (**i**). Note that a negative shRNA score suggests a decrease in cell proliferation/survival. *P* value was calculated using two-tailed Mann-Whitney test. **j**, Expression of c-MYC and a loading control β-actin in control and CARM1 knockout A1847 cells determined by immunoblot. Immunoblots are representative of two biologically independent experiments with similar results. **k**, B-I09 dose response curves in A1847 cells treated with CARM1 enzymatic activity inhibitor EZM2302 (20 μg/ml) or vehicle control. Please see Supplementary Figure 3d for validation of enzymatic activity inhibition. Data represent mean ± SEM, n = 4 biologically independent experiments. *P* values were calculated using a two-tailed *t* test. **l**, Expression of unspliced and spliced *XBP1* in the indicated high grade serous ovarian cancer (HGSOC) cell lines was determined by RT-qPCR analysis. And the ratio of spliced and unspliced *XBP1* was calculated accordingly in the indicated cell lines. Data represent mean ± SEM, n = 3 biologically independent experiments. *P* values were calculated using a two-tailed *t* test. **m**, Expression of CARM1, XBP1s and a loading control β-actin in control and CARM1 knockout A1847 cells treated with vehicle control or tunicamycin (5 μg/ml, 8h). Immunoblots are representative of three biologically independent experiments with similar results. **n**, Expression of XBP1s, IRE1α and a loading control β-actin in control and CRISPR-mediated CARM1 overexpressing EF027 and COV362 cells treated with vehicle control or tunicamycin (5 μg/ml, 8h). Immunoblots are representative of two biologically independent experiments with similar results. **o**, RT-qPCR analysis of *DDIT3* (encoding CHOP) mRNA expression in control and CARM1 knockout A1847 cells treated with vehicle control or tunicamycin (5 μg/ml, 8h). **p**, Same as k, but for CHOP and a loading control β-actin protein expression as determined by immunoblot analysis. Immunoblots are representative of two biologically independent experiments with similar results. **q**, Validation of CHOP knockdown by the indicated shRNAs in A1847 cells treated with vehicle control or tunicamycin (5 μg/ml, 8h). Immunoblots are representative of two biologically independent experiments with similar results. Data represent mean ± SEM, n = 3 biologically independent experiments. *P* values were calculated using a two-tailed *t* test.

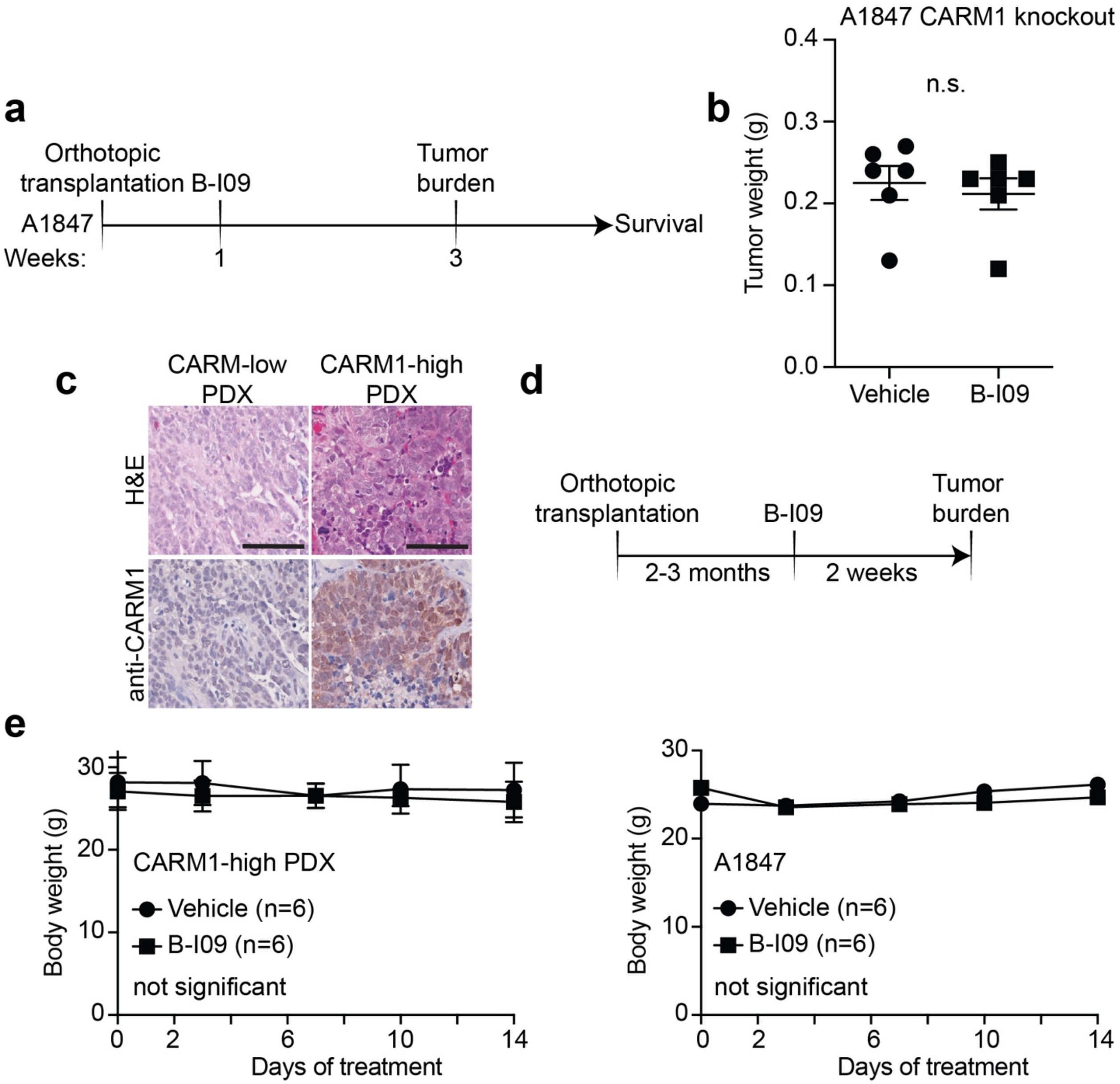

Supplementary Figure 5. CARM1 status correlates with response to IRE1α inhibitor B-I09 *in vivo*.

a, Schematic of experimental design for orthotopic xenograft models using control or CARM1 knockout A1847 cells. **b**, CARM1 knockout A1847 cells were unilaterally injected into the ovarian bursa sac of immunocompromised mice. Tumor-bearing mice were randomized and treated with vehicle control or B-I09 (50 mg per kg, 5 weekdays per week by i.p.) for 2 weeks. After treatment, tumors were surgically dissected and tumor weight was measured as a surrogate for tumor burden (n = 6 mice per group). *P* values were calculated using a two-tailed *t* test. **c**, Immunohistochemical (IHC) staining with an anti-CARM1 antibody in CARM1-low and CARM1-high PDXs. Pictures are representatives of two independent IHC experiments for each tumor with similar results. Scale bar = 100 μm. **d**, Schematic of experimental design for PDX mouse model was shown. **e**, Body weights of mice in indicated groups during vehicle control or B-I09 treatment. *P* values were calculated using a two-tailed t test.

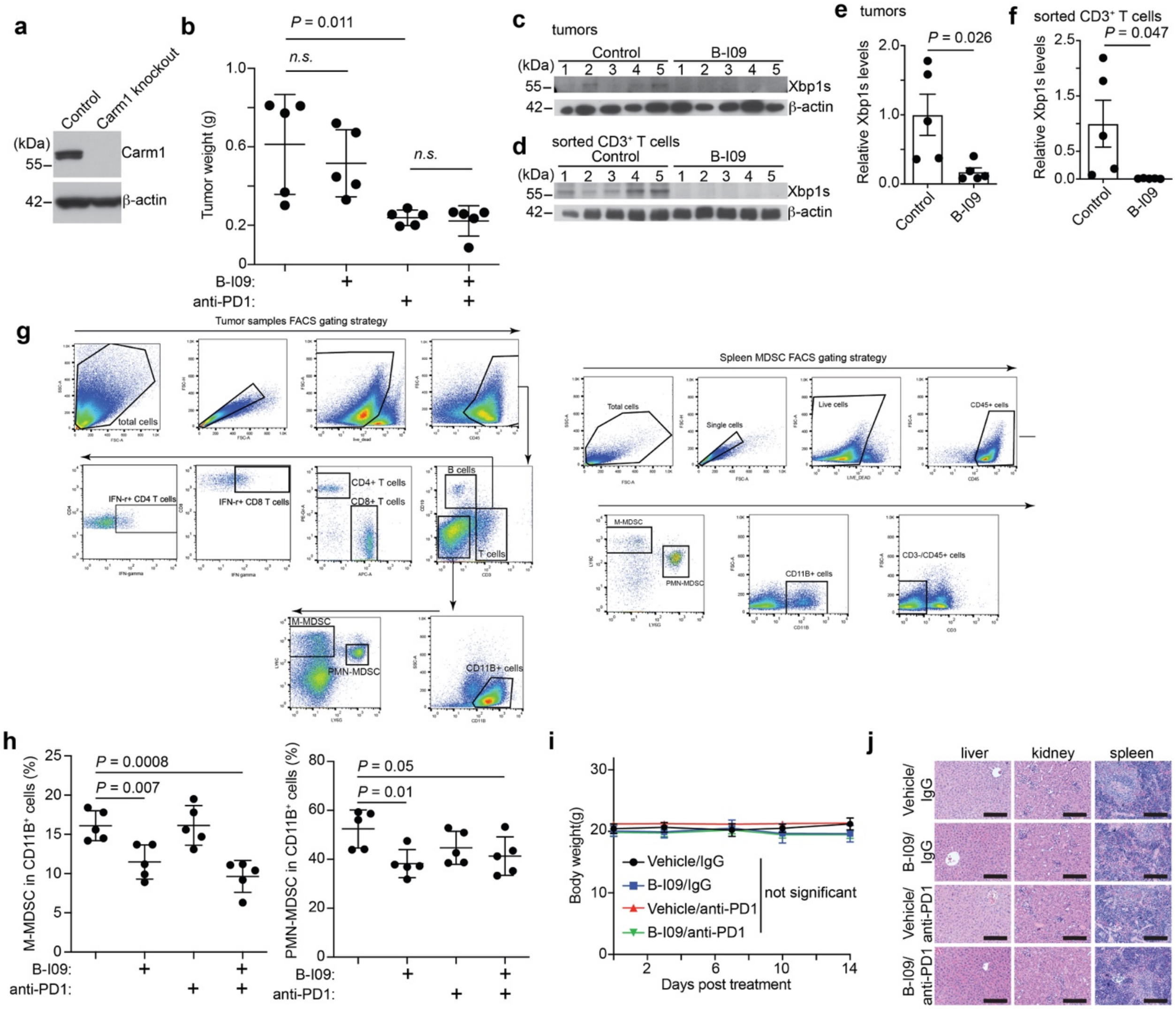

Supplementary Figure 6. B-I09 and anti-PD1 combination is well tolerated.
a, Carm1 and a loading control b-actin expression in control and Carm1 knockout UPK10 cells determined by immunoblot analysis. Immunoblots are representative of three biologically independent experiments with similar results. **b**, Mice bearing orthotopic tumors formed by Carm1 knockout UPK10 cells were randomized into four indicated treatment groups. The weights of tumors dissected from the indicated groups were measured as a surrogate for tumor burden (n=5 mice per group). *P* values were calculated using a two-tailed *t* test. **c-d**, Expression of Xbp1s and a loading control β-actin in tumors (**c**) or sorted CD3 positive T cells (**d**) in the treated mice bearing tumors formed by UPK10 cells was determined by immunblots (n=5 mice per group). Immunoblots are representative of two biologically independent experiments with similar results. **e-f**, Quantification of relative Xbp1s levels in **c-d** based on density of immunoblot bands and normalized against β-actin using NIH ImageJ software. Mice bearing orthotopic tumors formed by Carm1 knockout UPK10 cells were randomized into four indicated treatment groups. The weights of tumors dissected from the indicated groups were measured as a surrogate for tumor burden (n=5 mice per group). Data represent mean ± SEM, n = 3 biologically independent experiments. *P* values were calculated using a two-tailed t test. **g**, The gating strategy used for determining the percentage of the indicated immune cell populations. **h**, Changes in mononuclear myeloid-derived suppressor cells (M-MDSC) and polymorphonuclear

myeloid-derived suppressor cells (PMN-MDSC) induced by the indicated treatments in spleen were analyzed by flow cytometry. n = 5 mice per group. *P* values were calculated using a two-tailed *t* test. **i**, Body weights of mice in indicated groups during treatment (n = 6). *P* values were calculated using a two-tailed t test. **j**, Representative hematoxylin and eosin (HE) staining of liver, kidney and spleen from mice in indicated treatment groups. Pictures are representatives of two independent experiments for each tumor with similar results. Scale bar = 50 μm.

Supplementary Table 1. List of primers used in this study

**Primers for**	**Sequence (5'-->3')**
**RT-PCR:**	
human *HSPA5* RT-F	CAACGCCAAGCAACCAAAG
human *HSPA5* RT-R	TCCCTCTTATCCAGGCCATAA
human *DNAJB9* RT-F	TTGGCCATGAAGTACCACCC
human *DNAJB9* RT-R	AGCACTGTGTCCAAGTGTATCA
human *GAPDH* RT-F	GTCTCCTCTGACTTCAACAGCG
human *GAPDH* RT-R	ACCACCCTGTTGCTGTAGCCAA
human *DDIT3* RT-F	ACCAGGAAACGGAAACAGAG
human *DDIT3* RT-R	CTGTGCCACTTTCCTTTCATTC
**ChIP-qPCR:**	
human *HSPA5* ChIP-F	CAATGAACGGCCTCCAACG
human *HSPA5* ChIP-R	TCGGCTTATATACCCTCCCCC
human *DNAJB9* ChIP-F	TGGGGAAGCGTTTCGTGTAG
human *DNAJB9* ChIP-R	CTGGCACGCACCCTAATCTC
**Cloning:**	
CARM1 FL-F	AAAGAATTCATGGCAGCGG CGGCGGCGGC
CARM1 FL-R	AAAGCGGCCGCCTAGCTCCCGTAGTGCATGGT
CARM1 28-608-F	AAAGAATTCACCGTGTCGGTGTTCCCCGGC
CARM1 28-608-R	AAAGCGGCCGCCTAGCTCCCGTAGTGCATGGT
CARM1 1-140-F	AAAGAATTCATGGCAGCGG CGGCGGCGGC
CARM1 1-140-R	AAAGCGGCCGCCTCGCTGAACACAGACCGCT
CARM1 141-480-F	AAAGAATTCCGGACGGAGGAGTCTTCTGC
CARM1 141-480-R	AAAGCGGCCGCTGTGCCCGTGTATCTAAAGA
CARM1 481-608-F	AAAGAATTCACGCCCTCACCCCCACCCGGC
CARM1 481-608-R	AAAGCGGCCGCCTAGCTCCCGTAGTGCATGGT
CARM1 gRNA	AGCACGGAAAATCTACGCGG
