# Supplementary material for: Targeting the IRE1α/XBP1s pathway suppresses CARM1-expressing ovarian cancer
Source: Nat Commun. 2021 Sep 7;12:5321. doi: 10.1038/s41467-021-25684-3 (PMC8423755; doi:10.1038/s41467-021-25684-3)
Supplement: Supplementary file 3 — Description of Additional Supplementary Files [file 41467_2021_25684_MOESM3_ESM.pdf]

**Description of Additional Supplementary Files**

File Name: Supplementary Data 1
Description: List of 363 direct CARM1/XBP1s target genes upregulated by ER stress inducer tunicamycin

File Name: Supplementary Data 2
Description: List of 430 CARM1/XBP1s direct target genes that are downregulated by CARM1 knockout and XBP1 knockdown
