# Supplementary material for: Targeting the IRE1α/XBP1s pathway suppresses CARM1-expressing ovarian cancer
Source: Nat Commun. 2021 Sep 7;12:5321. doi: 10.1038/s41467-021-25684-3 (PMC8423755; doi:10.1038/s41467-021-25684-3)
Supplement: Supplementary file 6 — Reporting Summary [file 41467_2021_25684_MOESM6_ESM.pdf]

nature research

Corresponding author(s): Rugang Zhang

Last updated by author(s): Aug 11, 2021

Reporting Summary

Nature Research wishes to improve the reproducibility of the work that we publish. This form provides structure for consistency and transparency in reporting. For further information on Nature Research policies, see our Editorial Policies and the Editorial Policy Checklist.

Statistics

For all statistical analyses, confirm that the following items are present in the figure legend, table legend, main text, or Methods section.

n/a	Confirmed	
☐	☒	The exact sample size (*n*) for each experimental group/condition, given as a discrete number and unit of measurement
☐	☒	A statement on whether measurements were taken from distinct samples or whether the same sample was measured repeatedly
☐	☒	The statistical test(s) used AND whether they are one- or two-sided<br>*Only common tests should be described solely by name; describe more complex techniques in the Methods section.*
☒	☐	A description of all covariates tested
☒	☐	A description of any assumptions or corrections, such as tests of normality and adjustment for multiple comparisons
☐	☒	A full description of the statistical parameters including central tendency (e.g. means) or other basic estimates (e.g. regression coefficient) AND variation (e.g. standard deviation) or associated estimates of uncertainty (e.g. confidence intervals)
☐	☒	For null hypothesis testing, the test statistic (e.g. *F*, *t*, *r*) with confidence intervals, effect sizes, degrees of freedom and *P* value noted<br>*Give P values as exact values whenever suitable.*
☒	☐	For Bayesian analysis, information on the choice of priors and Markov chain Monte Carlo settings
☒	☐	For hierarchical and complex designs, identification of the appropriate level for tests and full reporting of outcomes
☐	☒	Estimates of effect sizes (e.g. Cohen's *d*, Pearson's *r*), indicating how they were calculated

Our web collection on statistics for biologists contains articles on many of the points above.

Software and code

Policy information about availability of computer code

Data collection	Flowjo (V10) was used for immune profiling. Images were analyzed using ImageJ (version:1.53f) software.
Data analysis	Prism 7 for Windows was used for calculating p values. bowtie, HOMER, bigWigAverageOverBed (UCSC toolbox) were used for cut-and-run analysis. Bowtie2, RSEM v1.2.12 and DESeq2 were used for RNA-sequencing analysis.

For manuscripts utilizing custom algorithms or software that are central to the research but not yet described in published literature, software must be made available to editors and reviewers. We strongly encourage code deposition in a community repository (e.g. GitHub). See the Nature Research guidelines for submitting code & software for further information.

Data

Policy information about availability of data

All manuscripts must include a data availability statement. This statement should provide the following information, where applicable:

- Accession codes, unique identifiers, or web links for publicly available datasets
- A list of figures that have associated raw data
- A description of any restrictions on data availability

The cut-and-run and RNA-seq data generated in this study have been deposited in the Gene Expression Omnibus (GEO) under accession GSE157118 (https://www.ncbi.nlm.nih.gov/geo/query/acc.cgi?acc=GSE157118). Source data are provided with this paper. TCGA HGSOC RNA-seq dataset was downloaded from cBioPortal (https://www.cbioportal.org/). Cancer Cell Line Encyclopedia RNA-seq data were downloaded from (https://sites.broadinstitute.org/ccle/datasets). hg19 was downloaded from UCSC (https://hgdownload.soe.ucsc.edu/downloads.html).

April 2020

Field-specific reporting

Please select the one below that is the best fit for your research. If you are not sure, read the appropriate sections before making your selection.

☒ Life sciences ☐ Behavioural & social sciences ☐ Ecological, evolutionary & environmental sciences

For a reference copy of the document with all sections, see nature.com/documents/nr-reporting-summary-flat.pdf

Life sciences study design

All studies must disclose on these points even when the disclosure is negative.

Sample size	No statistical method was used to predetermine sample size. Sample size was determined. Sample size was determined based on previous studies which have shown robust statistical power. Experiments were repeated 3 times experimentally unless otherwise stated for in vitro studies. At least 5 mice in each experimental group were used for in vivo experiments.
Data exclusions	There was no exclusion from the experiments.
Replication	All experiments in the manuscript were performed at least twice independently. Each experiment in vitro contained 3 independent replicates per sample. Each experiment in vivo was performed with n=5-10 mice/group. All attempts at replication were successful. Exact numbers of biologically independent experimental repetition are stated in the manuscript.
Randomization	Animals were randomly assigned to groups. PLA and IHC pictures are randomly taken on the slides and quantified the PLA signal and H-score, respectively.
Blinding	Investigators were blinded during data collection and analysis.

Reporting for specific materials, systems and methods

We require information from authors about some types of materials, experimental systems and methods used in many studies. Here, indicate whether each material, system or method listed is relevant to your study. If you are not sure if a list item applies to your research, read the appropriate section before selecting a response.

Materials & experimental systems

n/a	Involved in the study	
☐	☒	Antibodies
☐	☒	Eukaryotic cell lines
☒	☐	Palaeontology and archaeology
☐	☒	Animals and other organisms
☒	☐	Human research participants
☒	☐	Clinical data
☒	☐	Dual use research of concern

Methods

n/a	Involved in the study	
☐	☒	ChIP-seq
☐	☒	Flow cytometry
☒	☐	MRI-based neuroimaging

Antibodies

Antibodies used	For Western blotting, ChIP and PLA, the following primary antibodies were used: rabbit anti-CARM1 (Cell Signaling, #3379S), mouse anti-CARM1 (Cell Signaling, #12495), rabbit anti-XBP1 (Cell Signaling, #12782S), anti-cleaved PARP (Cell Signaling, #5625S), rabbit anti-lamin A/C (Cell Signaling, #2032S), mouse anti-CHOP (Cell Signaling, #2895), mouse anti-beta-actin (Sigma, #A5316), rabbit anti-flag-M2 (Cell Signaling, #2368S), rabbit anti-H3R17me2a (Abcam, #ab8284), mouse anti-histone H3 (Cell Signaling, #14269S), rabbit anti-XBP1 (Novus Biologicals, # NBP1-77681). For flow cytometry, the following primary antibodies were against: mouse CD45 (Biolegend, #103147), mouse CD3(BD, #552774), mouse CD4 (Biolegend, #100516), mouse CD8 (Biolegend, #100708), mouse anti-CD11C (Biolegend, #117324), mouse CD11B (Biolegend, #101259), mouse B220 (Biolegend, #103227), mouse CD19 (Biolegend, #115523), mouse Ly6C (Biolegend, #128026), mouse Fcg (BD, #553142) and mouse Ly6G (Biolegend, #127639). For IHC, anti-cleaved caspase 3 (Cell Signaling, #9661), and anti-Ki67 (Cell Signaling, #9449) were used.
Validation	Information of each antibody is available on the manufacturer's website.<br>The following antibodies which are validated by the manufacturer for western blots using human cell lysates were used in this manuscript for western blots of human cell lysates: rabbit anti-CARM1 (Cell Signaling, #3379S), rabbit anti-XBP1 (Cell Signaling, #12782S), anti-cleaved PARP (Cell Signaling, #5625S), rabbit anti-lamin A/C (Cell Signaling, #2032S), mouse anti-beta-actin (Sigma, #A5316), rabbit anti-flag-M2 (Cell Signaling, #2368S), rabbit anti-H3R17me2a (Abcam, #ab8284), mouse anti-histone H3 (Cell Signaling, #14269S), rabbit anti-XBP1 (Novus Biologicals, # NBP1-77681), mouse anti-CHOP (Cell Signaling, #2895). rabbit anti-CARM1 (Cell Signaling, #3379S), rabbit anti-XBP1 (Cell Signaling, #12782S), mouse anti-CHOP (Cell Signaling, #2895) were validated by either knockdown or knockout in this study.<br><br>The following antibodies which are validated by the manufacturer for western blots using mouse cell lysates were used in this

manuscript for western blots of mouse cell lysates: mouse anti-CARM1 (Cell Signaling, #12495), mouse anti-beta-actin (Sigma, #A5316), rabbit anti-XBP1 (Novus Biologicals, # NBP1-77681).

For immunoprecipitation using human lysates, mouse anti-CARM1 (Cell Signaling, #12495) was validated for IP and shown in manufacturer's website. In addition, mouse anti-CARM1 (Cell Signaling, #12495) was validated using CARM1 knockout.

For PLA in human cells, rabbit anti-XBP1 (Novus Biologicals, # NBP1-77681) and mouse anti-CARM1 (Cell Signaling, #12495) were validated for IF by the manufacturers.

For ChIP using human lysates, rabbit anti-CARM1 (Cell Signaling, #3379S), anti-H3R17me2a (Abcam, #ab8284) and rabbit anti-XBP1 (Novus Biologicals, # NBP1-77681) were validated by CARM1 knockout and XBP1 knockdown, respectively.

For IHC in mouse and human tumors, anti-cleaved caspase 3 (Cell Signaling, #9661), and anti-Ki67 (Cell Signaling, #9449) were validated for IHC by the manufacturers.

For flow cytometry of mouse cells, antibodies against: mouse CD45 (Biolegend, #103147), mouse CD3(BD, #552774), mouse CD4 (Biolegend, #100516), mouse CD8 (Biolegend, #100708), mouse anti-CD11C (Biolegend, #117324), mouse CD11B (Biolegend, #101259), mouse B220 (Biolegend, #103227), mouse CD19 (Biolegend, #115523), mouse Ly6C (Biolegend, #128026), mouse Fcg (BD, #553142) and mouse Ly6G (Biolegend, #127639) were validated by the manufacturers and published literatures.

Eukaryotic cell lines

Policy information about cell lines

Cell line source(s)	HEK293FT was purchased from Thermo Fisher Scientific (R70007); OVCAR3, OVCAR4, CAOV3 cell lines were purchased from ATCC; PEO4, PEO1, COV362, COV318, ID8 cell lines were purchased from Sigma; OVSAHO, Kuramochi cell lines were purchased from JCRB; A1847 and OVCAR10 cell lines were gifts from Dr.A. Godwin, Fox Chase Cancer Center (currently at University of Kanas Cancer Center); OVCAR8 was purchased from EZ Biosystem; UPK10 cell line was obtained as previously published.
Authentication	Cell lines were re-authenticated by The Wistar Institute's Genomics Facility using short tandem repeat profiling using AmpFLSTR Identifiler PCR Amplification kit (Life Technologies).
Mycoplasma contamination	Regular Mycoplasma testing was performed using LookOut Mycoplasma PCR detection (Sigma). All cell lines applied in this study were tested negative for Mycoplasma.
Commonly misidentified lines (See ICLAC register)	No commonly misidentified cell lines used.

Animals and other organisms

Policy information about studies involving animals; ARRIVE guidelines recommended for reporting animal research

Laboratory animals	6-week female NSG mice were purchased from Wistar Institute Animal Facility. 6-week female C57BL/6 mice were purchased from Charles River Laboratories.
Wild animals	This study did not involve wild animal.
Field-collected samples	No field-collected samples were used in this study.
Ethics oversight	Animal protocols were approved by the Institutional Animal Care and Use Committee (IACUC) of The Wistar Institute.

Note that full information on the approval of the study protocol must also be provided in the manuscript.

ChIP-seq

Data deposition

☒ Confirm that both raw and final processed data have been deposited in a public database such as GEO.

☒ Confirm that you have deposited or provided access to graph files (e.g. BED files) for the called peaks.

Data access links<br>*May remain private before publication.*	https://www.ncbi.nlm.nih.gov/geo/query/acc.cgi?acc=GSE157118<br>Private token: exuxcaoctdubtgn
Files in database submission	CARM1.fastq<br>input.fastq<br>CARM1.ctr.fastq<br>CARM1.TM.fastq<br>XBP1.ctr.fastq<br>XBP1.TM.fastq<br>input.ctr.fastq<br>input.TM.fastq<br>CARM1.bw

input.bw
CARM1.ctr.bw
CARM1.TM.bw
XBP1.ctr.bw
XBP1.TM.bw
input.ctr.bw
input.TM.bw

Genome browser session (e.g. UCSC)

https://genome.ucsc.edu/cgi-bin/hgTracks?db=hg19&hgsid=918161581_mZi2hZLWUQaBrN9zuaUJtunSaU1D

Methodology

Replicates

CARM1, XBP1 ChIP-seq was done in one replicate in Tunicamycin treated or untreated condition.

Sequencing depth

sample reads
CARM1 41,477,221
input 62,222,136
CARM1.ctr 50,969,786
CARM1.TM 52,854,349
XBP1.ctr 59,401,754
XBP1.TM 65,123,604
input.ctr 47,363,092
input.TM 49,300,641

Antibodies

rabbit anti-CARM1 (Cell Signaling, #3379S, lot: 1), rabbit anti-XBP1 (Cell Signaling, #12782S, lot: 4)

Peak calling parameters

HOMER: findPeaks -center -style factor

Data quality

Peaks that passed FDR<5% with at least 4-fold over control threshold were considered significant.

Software

bowtie, HOMER

Flow Cytometry

Plots

Confirm that:

☒ The axis labels state the marker and fluorochrome used (e.g. CD4-FITC).

☒ The axis scales are clearly visible. Include numbers along axes only for bottom left plot of group (a 'group' is an analysis of identical markers).

☒ All plots are contour plots with outliers or pseudocolor plots.

☒ A numerical value for number of cells or percentage (with statistics) is provided.

Methodology

Sample preparation

Tumor was minced into small (1 to 2 mm) pieces and digested with 1 mg/mL collagenase IV (Sigma-Aldrich, #C5138), 0.1 mg/mL Hyaluronidase (Sigma-Aldrich, #H6254) and 0.01 mg/mL deoxyribonuclease I (Sigma-Aldrich, #D5025). The cells were sequentially filtered through 45um cell strainer. Single-cell suspensions were prepared, and red blood cells were lysed using ACK Lysis Buffer (Thermo Fisher, #A1049201). Tumor infiltrated lymphocytes (TILs) were followed by viability staining (Thermo Fisher, #L34957). Before antibody staining, cells were blocked by an anti-Fcg receptor antibody (BD, #553142) and then surface staining was performed in FACS buffer (3% FBS in PBS) with fluorochrome-conjugated antibodies against: mouse CD45 (Biolegend, #103147), mouse CD3(BD, #552774), mouse CD4 (Biolegend, #100516), mouse CD8 (Biolegend, #100708), mouse CD11C (Biolegend, #117324), mouse CD11B (Biolegend, #101259), mouse B220 (Biolegend, #103227), mouse B cell marker CD19, mouse CD19 (Biolegend, #115523), mouse Ly6C (Biolegend, #128026) and mouse Ly6G (Biolegend, #127639).

For IFNg staining, freshly isolated cells in RPMI with L-glutamine (Thermo Fisher, # 25030149) (supplemented with 10% FBS, 1x non-essential amino acids, 1x streptomycin and penicillin, 50 nM/mL b-mercaptoethanol) were stimulated with cell activation cocktail with brefeldin A (Biolegend, #423303) overnight, then the cells in the supernatant were collected for the indicated surface staining, and then intracellular IFNg staining was performed using an anti-mouse IFNg antibody (Biolegend, # 505840).

Instrument

BD LSR II and Canto II Flow Cytometer

Software

FlowJo (V10) software (Tree Star, Inc)

Cell population abundance

no sorting was performed with the flow cytometer.

Gating strategy

cell debris were removed using FSC-A and SSC-A. Doublets were removed from total population using FSC-A and FSC-H. Live

Gating strategy	cells were gated by Live/Dead AmCyan-A. Single staining were used for compensation calculation. Positive population were gated based on comparing unstained samples and single stained sample as described in the Supplementary Fig.6g.

☒ Tick this box to confirm that a figure exemplifying the gating strategy is provided in the Supplementary Information.
