# Supplementary material for: Targeting the IRE1α/XBP1s pathway suppresses CARM1-expressing ovarian cancer
Source: Nat Commun. 2021 Sep 7;12:5321. doi: 10.1038/s41467-021-25684-3 (PMC8423755; doi:10.1038/s41467-021-25684-3)

Source data: uncropped version of gels.blots

Figure 2

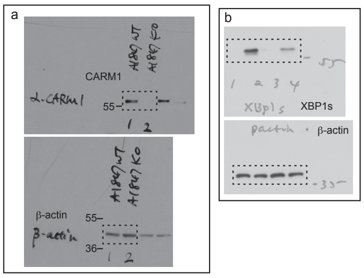

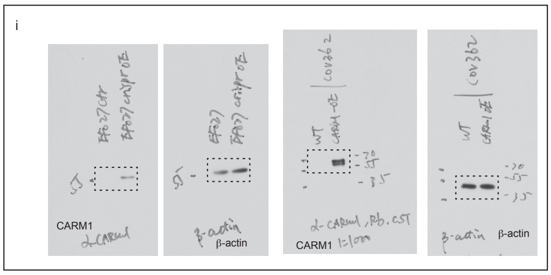

Figure 4

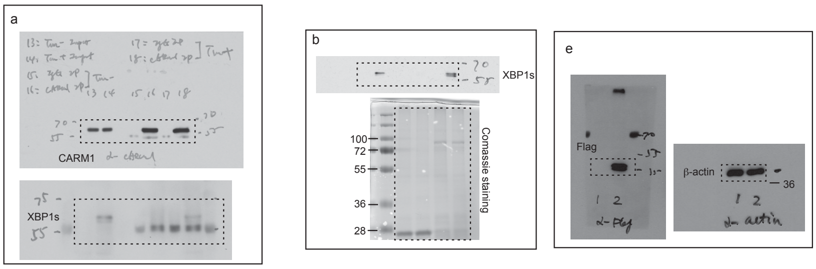

Figure 5

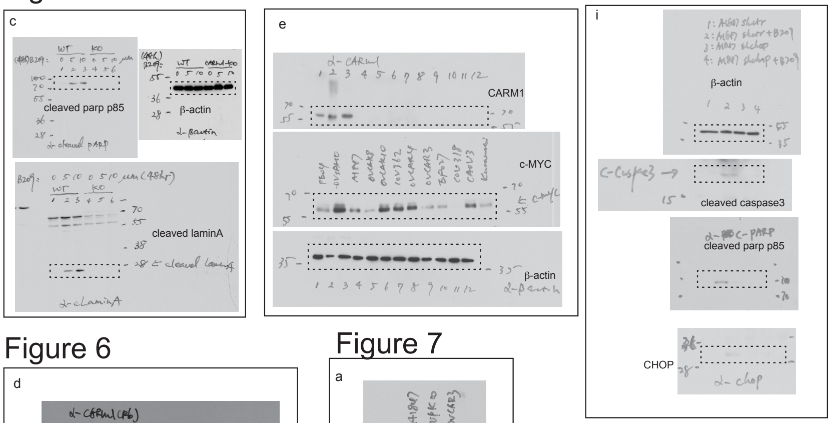

Figure 6

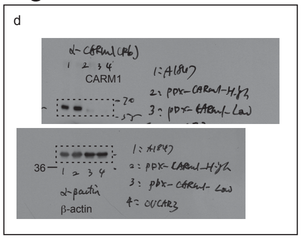

Figure 7

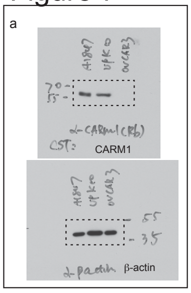

Supplementary Figure 6

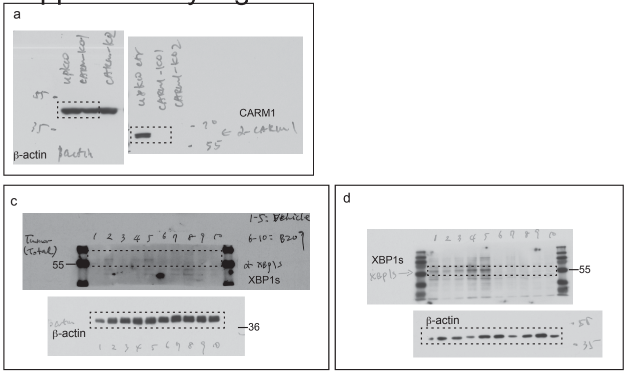

Supplementary Figure 1

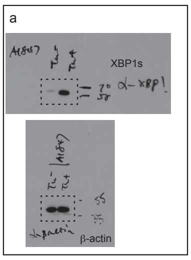

Supplementary Figure 2

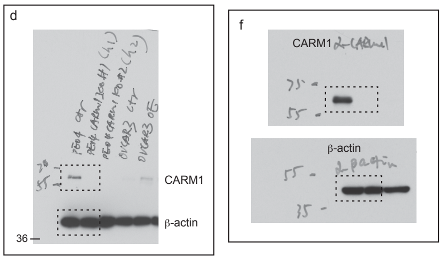

Supplementary Figure 3

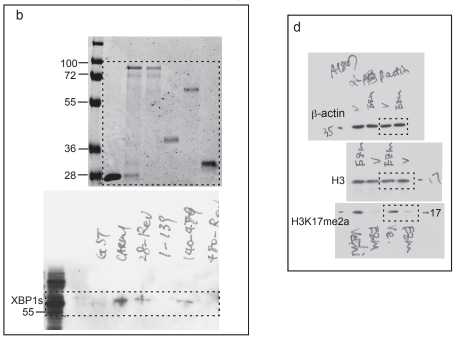

Supplementary Figure 4

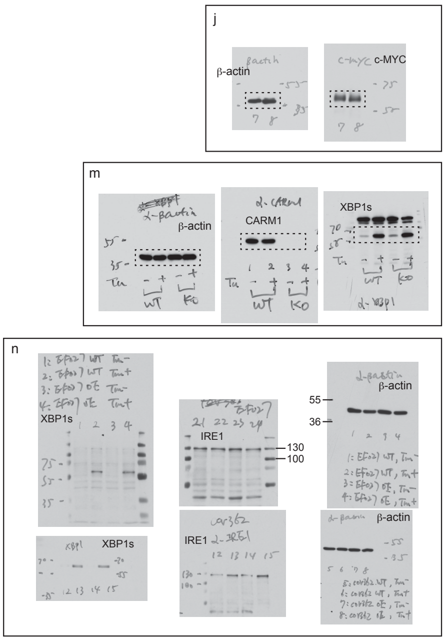

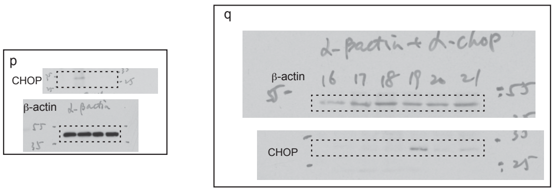

Supplement: Supplementary file 7 — Source Data [file 41467_2021_25684_MOESM7_ESM.zip › Source Data for Immunoblots.pdf]
